# Controlling Directed Protein Interaction Networks in Cancer

**DOI:** 10.1038/s41598-017-10491-y

**Published:** 2017-09-04

**Authors:** Krishna Kanhaiya, Eugen Czeizler, Cristian Gratie, Ion Petre

**Affiliations:** 1 Computational Biomodeling Laboratory, Turku Centre for Computer Science, and Department of Computer Science, Åbo Akademi University, Turku, 20500 Finland; 2 National Institute for Research and Development for Biological Sciences, Bucharest, Romania

## Abstract

Control theory is a well-established approach in network science, with applications in bio-medicine and cancer research. We build on recent results for structural controllability of directed networks, which identifies a set of driver nodes able to control an a-priori defined part of the network. We develop a novel and efficient approach for the (targeted) structural controllability of cancer networks and demonstrate it for the analysis of breast, pancreatic, and ovarian cancer. We build in each case a protein-protein interaction network and focus on the survivability-essential proteins specific to each cancer type. We show that these essential proteins are efficiently controllable from a relatively small computable set of driver nodes. Moreover, we adjust the method to find the driver nodes among FDA-approved drug-target nodes. We find that, while many of the drugs acting on the driver nodes are part of known cancer therapies, some of them are not used for the cancer types analyzed here; some drug-target driver nodes identified by our algorithms are not known to be used in any cancer therapy. Overall we show that a better understanding of the control dynamics of cancer through computational modelling can pave the way for new efficient therapeutic approaches and personalized medicine.

## Introduction

The main cause of cancer is genetic and epigenetic alterations, which allow normal cells to over-proliferate as tumour cells^1^. Most of these alterations contribute to various cancer dysregulated signal transduction pathways, which control essential cell processes such as growth factor, differentiation and survival^1^. Through signal transduction processes, these tumour cells develop as malignant cells^1, 2^; this complex information process is transmitted through protein-protein interactions (PPIs)^1^. Proteins act as the vehicles of these signals, while the interactions among them influence the velocity of the information flow. For instance, PPIs are directly regulating the phosphorylation of serine/threonine residues^3^, and the same process is used by tumour necrosis factor to convey signals from the receptor to their downstream targets^3^. Also, the transforming growth factor-*β* (TGF *β*) employs PPIs to convey signals to activate its targets^3^. TGF *β* interacts also with other signaling pathways^4^ and creates a complex web in cancer signaling. It has been shown that TGF *β* also regulates various kinase cascades such as the mitogen-activated protein kinase (MAPKs) ERK, the p38 MAPK pathways, the Jun N-terminal kinase (JNK), the PI3K kinase, the PP2A phosphatases and the Rho family members^5, 6^. Furthermore, by using docking proteins and protein interaction domains, the receptor tyrosine kinases (RTKs) recruits targets to the receptor^3^. These protein domains mediate a series of intra-molecular interactions during the downstream of RTKs and re-wire the signaling networks^7^. Usually, RTK modules are highly mutated and over-expressed in cancer, which effectively leads its signaling to escalate the progression of tumours^8^. Also, RTKs help to build robust cancerous signalling networks and signal to other tumor cells to form similar networks^9^. These studies show that to comprehensively understand the specificity in signaling networks, we have to understand how distinct pathways communicate with each other and how proteins of one pathway make interactions with related signaling components. A network approach over the cancer’s signal transduction dynamics gives us the tools to provide a better understanding of the various information-processing abilities employed during the molecular alteration of the cancerous cells^10^.

In human diseases, both associated and non-associated diseased proteins interact with one-another to create disease modules^11^, and pave the way towards a layered configuration and understanding of these complex diseases^12^. Previous studies have shown that networks associated with the same disease family, as well as with common phenotypes tend to contain significant similarities between their disease modules^13^. Disease proteins produce some common tendencies, such as: inside and outside interactions of modules through PPIs, co-expression in specific tissues, as well as high expression correlations^14^. Uncovering these disease-specific interactions is essential not only in demonstrating the complex molecular mechanism inside these networks, but also in providing an inside-view of the dysfunctional signaling transduction processes within these networks. All these examples illustrate that a network approach toward disease analysis could provide significant new insights into disease-gene identifications, as well as it could open new approaches towards network-based therapeutic tools, targeting entire disease modules together instead of individual elements^15^. The current system-based understanding of biological processes has already showed that due to the various overlaps of signaling pathways, proteins participating in multiple pathways build robust inter-pathways connections. Therefore multi-target drugs can inhibit multiple proteins and can thus increase the chance of effective treatments^16^. In turn, targeting single proteins can damage the connection of multi-cellular functions and delay the recovery of disease^16–18^. However, in many diseases, the relationship between the various drug targets and the associated disease proteins is still vague. This opens a new door of investigation for finding rational disease control mechanisms by use of the currently available drug-target proteins. Essential proteins are of central interest in such investigations, in identifying novel targets for therapeutics^19^; there is already evidence that targeting survivability-essential proteins in cancer can lead to novel therapies^20, 21^. Proteins are consider survivability-essential (in short, essential) in cancer if their suppression leads to the death of the cancer cells^22^. Cancer essential proteins can be found in specific cell lines and often induce oncogenesis^21^.

Network biology, with the help of computational modeling, has revolutionized the human diseasome research and paved the way towards the development of new therapeutic approaches and personalized medicine^23^. This is why, in the last couple of decades, network science has been constantly in the focus of biological research, where scientists try to understand the dynamics and control features of various complex bio-chemical networks in association with matching experimental findings^10^. Recent work on network controllability has shown that full controllability and reprogramming of inter-cellular networks, which assumes the driving of the complete network from any initial state to any desired final state, can be achieved through a minimum number of control targets^23, 24^. However, the computer-based experimental tests of Liu *et al*.^25^ suggest that achieving full control over gene regulatory networks is rather demanding, requiring sometimes up to 80% of the nodes to be directly controlled. This makes the approach impractical in a medical setup where the external control is to be implemented through administering drugs. Another approach by Wuchty *et al*.^26^ focusses on the so-called Minimum Dominating Sets (MDS) for controlling the dynamics of a protein interaction network. An MDS is a minimal set of nodes within a network with the property that all the nodes in the network are directly connected to at least one element in the MDS. The conceptually-different assumption here is that any node in the network can be used to control all of its direct neighbours, i.e., those nodes in the network with whom there is a direct interaction.Within this framework, Wuchty *et al*.^26^ showed that the MDSets are enriched with essential, cancer-related and virus-targeted proteins, which are acting as bottlenecks for various essential cell processes. Based on the study from^27^ and considering essential MDSets (e-MDSets), Khuri *et al*.^27^ showed that e-MDSet proteins have predominantly more connections in networks than any other sets of proteins, and can be vital for network control. Another framework for network control based on feedback loops (both negative and positive) showed that these loops play a major role in signaling transduction networks by causing various oscillations and switching of signals^27^. What is missing from these approaches, that we instead focus on, is a direct targeting of survivability-essential genes and the practicalities of implementing it through a combinatorial drug-target approach.

In this article we use target controllability for the analysis of specific signal transduction cancer networks, focusing on cancer type specific essential proteins as our target nodes and on drug-target proteins of FDA-approved drugs as our driver nodes. In particular, we investigate the breast, pancreatic, and ovarian cancer, where the cancer essential genes to be used as controlling targets are specific to the MDA-MBD-231, HPAF-II and OV-90 cell lines, respectively. We develop a general computational model based on directed networks, that aims to find specific paths from the set of potential driver nodes to the set of targets. Generalizing a similar result for target controllability, we prove that the algorithmic problem of minimizing the size of the controlling set while restricting the search to a subset of potential, drug targetable, driver nodes is algorithmically hard (i.e., NP-hard). Considering the three STN/PPI networks analyzed in this study, we report on the total number of driver nodes needed to control the cell line specific essential protein targets, the number/list of drug targetable driver nodes, and on some interesting topological properties of the driver nodes in all of these networks. Also, we analyze the robustness of our predictions for both false-positives and false-negatives for the three networks above. In particular, we analyze the outcome differences when a small number of random protein signalling interactions are removed (or added) to the networks.

## Results

### Controlling PPI signaling transduction networks in cancer

To determine the controllability of essential proteins we analyzed three cancer networks: breast, pancreatic and ovarian cancers, see Table S2. The size of the PPI networks that we generated based on^28^ ranges roughly between 900–1600 nodes and between 1500–2500 edges, see Table 1.

**Table 1:** Full controllability of three cancer network.

Network	Nodes	Edges	Full control: driver (drug-targ.) nodes	Full control: % driver (% drug-targ.) nodes
Breast	1415	2532	962 (210)	68% (15%)
Pancreatic	991	1569	690 (472)	70% (48%)
Ovarian	1047	1643	736 (432)	70% (41%)

The columns represent the following information per cancer network: the total number of nodes in the network (Nodes), the number of connections (Edges), the controlling set for the entire network (Full control), the % of the controlling set vs. the whole network (Full control %).

We first computed the minimum set of nodes controlling the entire network, based on the algorithm for full controllability in ref. 25. We found in all three cases that around 70% of all the nodes have to be directly controlled in order to gain control over the whole network, Table 1. This is in accordance with previous results of ref. 25 for different types of gene regulatory networks and confirms that full controllability is impractical in cancer medicine.

**Figure 1:**
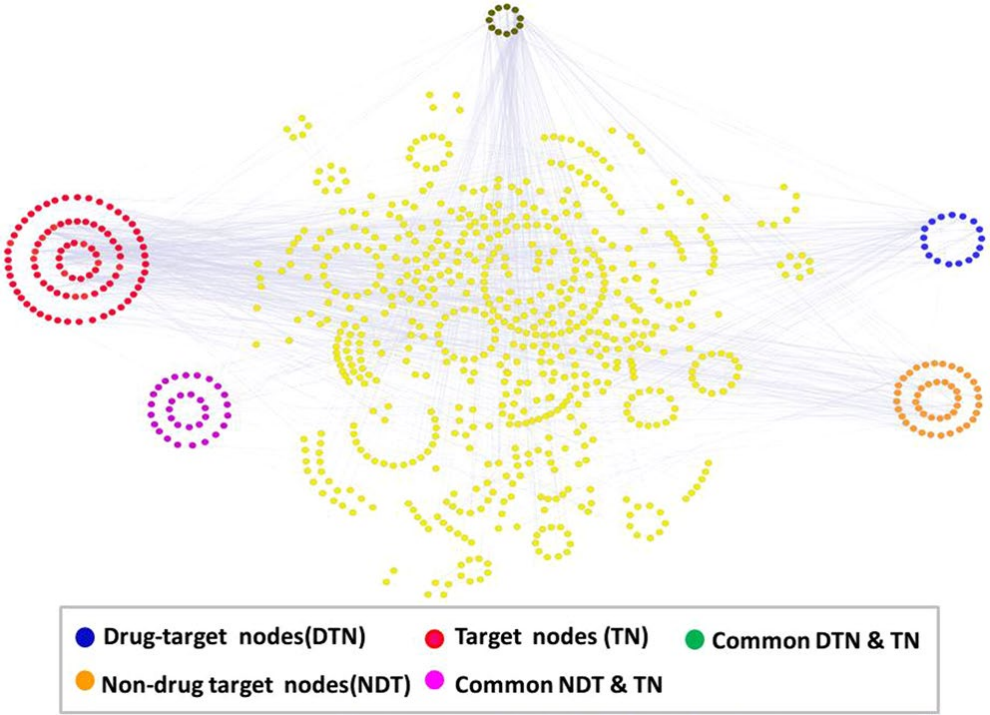
Pancreatic cancer PPI network. A network view of the pancreatic cancer PPI network. The network contains 90% of the total network nodes, while the remaining part of the network containing isolated nodes. The drug-target nodes (DTN) are shown in dark blue, target nodes (TN) are in maroon, nodes that are both in DTN and TN are shown in dark green, non-drug target nodes (NDT) are in light orange, and nodes that both in NDT and TN are in magenta.

**Figure 2:**
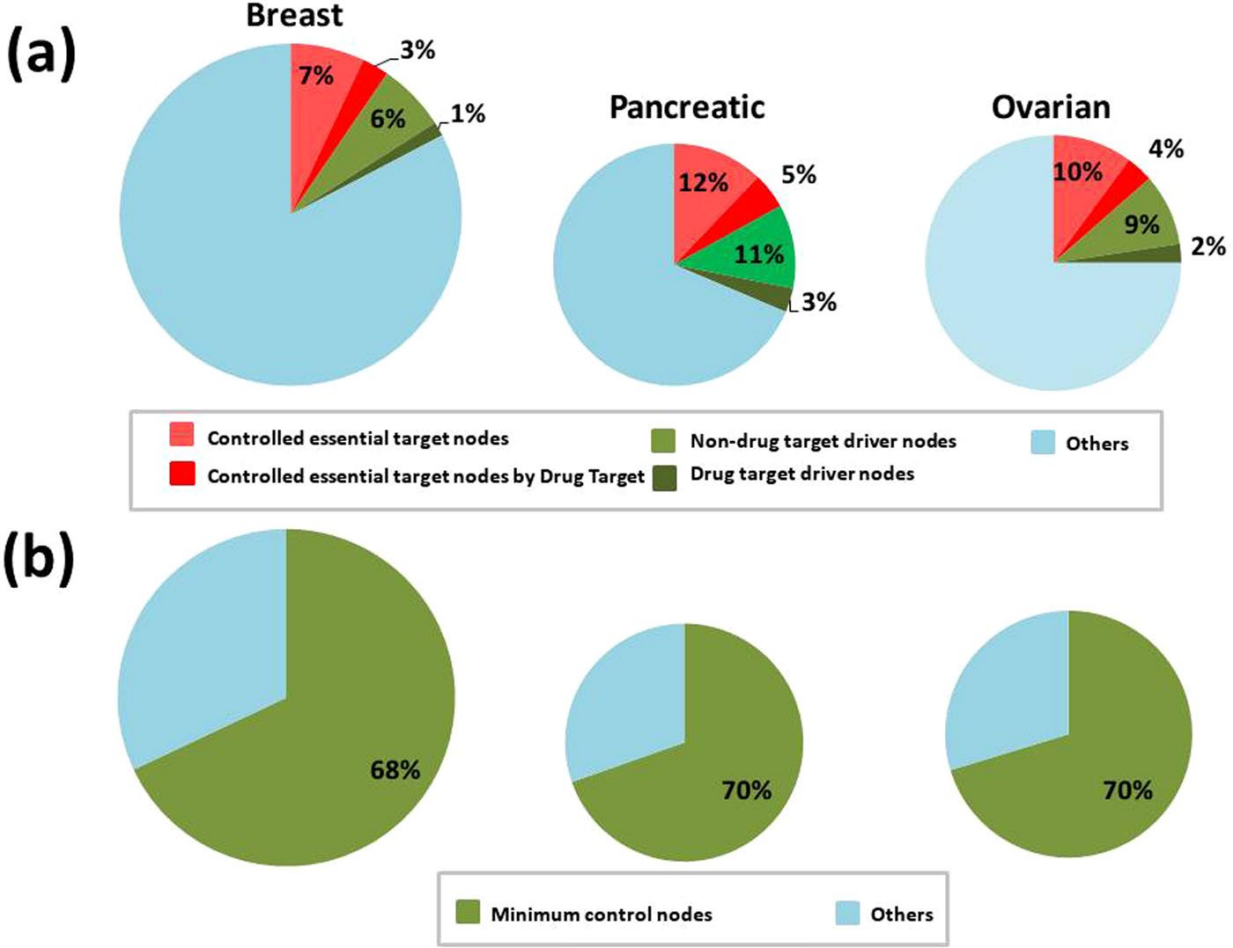
Controlling of cancer networks. The radius of the circles is proportional with the number of nodes in the networks. (**a**) The percentage of controlled target nodes by drug-target nodes and non drug-target nodes, w.r.t. the total number of nodes. (**b**) Required minimum control nodes for the control of the whole cancer networks.

We then considered a set of cancer type-specific essential proteins based on ref. 29 and computed the sets of nodes that are enough to (target) control these essential proteins, see Table S3. We applied both the generic algorithms of ref. 23 (whose search we improved through a new heuristic strategy according to ref. 30) and our algorithm maximizing the use of drug-targetable nodes as driver nodes, aiming to make the results more practical, see Supplementary Note S8: for more details of this approach. The results are summarized in Table 2; the cancer PPI networks are graphically described in Fig. 1 for pancreatic cancer, in Fig. S1 for breast cancer, and in Fig. S2 for ovarian cancer. We found that the number of driver nodes needed for the control of essential proteins is much smaller, ranging between 6–14% of the total number of nodes in the network, Fig. 2, depending on the cancer type and on the algorithms used in the computation. Our algorithms also found sets of driver nodes containing many (19–32) drug targetable nodes, Table 2, drastically improving the applicability of this approach.

**Table 2:** Essential gene-targeted controllability of three cancer networks.

Network	Nodes	Edges	Targets: #(%)	Min target control: driven (drug_tar)	Min target control(%): driven (drug_tar)	Drug_oriented target control: driven (drug_tar)	Drug_oriented target control(%): driven (drug_tar)
Breast	1415	2532	135 (9.5%)	94 (1)	6.6% (0%)	110 (19)	7.7% (1.3%)
Pancreatic	991	1569	168 (17%)	131 (9)	13.2% (0.9%)	143 (32)	14.4% (3.2%)
Ovarian	1047	1643	140 (13%)	111 (6)	10.6% (0.5%)	120 (25)	11.4% (2.3%)

The columns represent the following information per cancer network: the total number of nodes in the network (Nodes), the number of connections (Edges), the number (and percentage) of target proteins (Targets), the minimal controlling set of the target proteins (Min target control) including enclosed drug-target (drug_tar) proteins, percentage (vs. the whole network) of the minimal controlling set (Min target control (%)) including enclosed drug-targets, the drug-oriented controlling set (Drug-oriented target control) including enclosed drug-target (drug_tar) proteins, and the percentage (vs. the whole network) of the drug-oriented controlling set (Drug-oriented target control(%)) including enclosed drug-targets (drug_tar).

These results portray a highly advantageous situation. The ultimate objective of our use of structural controllability on cancer disease networks is to be able to control the cancer evolution and drive it towards a downfall. This can be achieved by gaining control over the entire network, an approach which seems to require an excessive direct intervention over 68%, 69.6%, and 70.3% of the nodes in the network, i.e., 962, 690, and 736 of the nodes from the breast, pancreatic and ovarian cancer networks, respectively, Fig. 2 and Table 1. In contrast to the previous situation, we aimed in achieving a control over a subset of nodes, specific to each individual cancer network, which is known to have an overwhelming effect over that cancer’s survivability. Controlling these focused targets can be done much more efficiently than controlling the whole network, requiring a direct intervention over 6.6% (94 nodes) 13.2% (131 nodes) and 10.6% (111 nodes) of the breast, pancreatic and ovarian cancer networks, respectively. Thus, we obtain up to a 10 fold decrease in the control effort, Fig. 2, while maintaining a high likelihood of an overall similar effect. Moreover, the enhanced variant of our algorithm aiming to maximise the use of drug-target proteins as drivers for network controllability found between 19 and 32 drug-targets (as opposed to 1–9 drug-targets in the non-enhanced version of the algorithm) that are predicted to influence cancer type-specific survivability-essential proteins. In fact, any non-empty subset of these drug-targets can control some of the essential genes in the network, which gives many drug combination suggestions for therapeutical purposes. This increases the practical applicability of our approach.

### Robustness of the results to false positives and false negatives

We analyzed the robustness of our structural target control algorithm with preferential operators for both false negatives and false positives. False negatives are particularly concerning as errors, as they stand for incomplete knowledge, which is a de-facto situation of most bio-medical databases; such databases could be subject to updates once per year, or sometimes even monthly. We proved mathematically, see Supplementary Note S9: that our algorithm is robust to false negatives: a set of driver nodes controlling a given set of target nodes will continue to control it regardless of how many additional interactions are added to the network. Naturally, with more interactions (i.e., more data), a smaller set of driver nodes may be found to control the same set of target nodes.

We analyzed the robustness of our algorithm to false positives through computer simulations. We focused on the overall prediction of drug-targets. Given a PPI network, a set of essential proteins, and a set of drug-targets (called more generally *preferential operators* in Supplementary Note S8), we define the *pool of solutions* as the set of all drug targets reported by the algorithm through 70 independent runs. We also define the *pool of recurrent solutions* as the set of those drug targets reported by the algorithm in at least 50% of the runs.

To estimate the impact of false positives on our results, we removed 5% of the edges, selected randomly from the network. We collected the pool of solutions and of recurrent solutions on the reduced PPI network through 70 independent runs. We repeated this for 10 independently reduced networks. For the numerical analysis of the similarity between the pool of solutions of the full network and that of the reduced networks we introduced two measures of robustness, and computed them independently for each of the 10 experiments. The *output similarity measure (output s.m.)* is the ratio between the size of the intersection and of the union of the pool of solutions of the full and the reduced network. This gives a measure of how much the set of predicted drug targets changes in the presence of false positives. The *core similarity measure (core s.m.)* is the ratio between the size of the intersection and of the union of the pool of recurrent solutions of the full and the reduced network. This gives a measure of how much the set of frequently predicted drug targets changes in the presence of false positives. The results, detailed in Table 3, show a relatively small change in the output of our algorithm in the presence of false positives. We conclude that our algorithm is featuring a strong robustness also for the case of false positives.

**Table 3:** Solution pool similarity between the normal and the 5% randomly reduced networks.

		*K*_1_	*K*_2_	*K*_3_	*K*_4_	*K*_5_	*K*_6_	*K*_7_	*K*_8_	*K*_9_	*K*_10_
*Breast	Output s.m.	0.833	0.897	0.935	0.934	0.917	0.901	0.885	0.935	0.685	0.928
	Core s.m.	0.845	0.873	0.884	0.925	0.875	0.854	0.872	0.908	0.731	0.909
*Pancreatic	Output s.m.	0.874	0.907	0.894	0.905	0.915	0.926	0.91	0.883	0.887	0.897
	Core s.m.	0.838	0.709	0.695	0.896	0.666	0.896	0.815	0.874	0.878	0.802
*Ovarian	Output s.m.	0.908	0.938	0.921	0.878	0.904	0.88	0.956	0.854	0.913	0.914
	Core s.m.	0.888	0.927	0.902	0.742	0.714	0.709	0.775	0.725	0.915	0.773

As a final robustness experiment performed on the (un-reduced) networks generated for breast, pancreatic and ovarian cancer, we looked at the frequency with which the set of drug-targets outputted by our algorithm within the minimal solution(s) (e.g., reported in column 7 of Table 2) appeared in any of the multiple independent runs of the algorithm (on the same networks). Thus, we computed the ratio between the number of those drug-targets appearing in both the minimal solution and in at least 66% of the solution sets of the other multiple runs (on the same network), over the total number of drug-targets appearing in the minimal solution. The results, 0.82 for breast, 0.76 for pancreatic, and 0.71 for ovarian cancer, show that these minimal solutions are highly representative for the entire solution pool of operators.

### Topological properties of drug target proteins and of essential proteins

**Figure 3:**
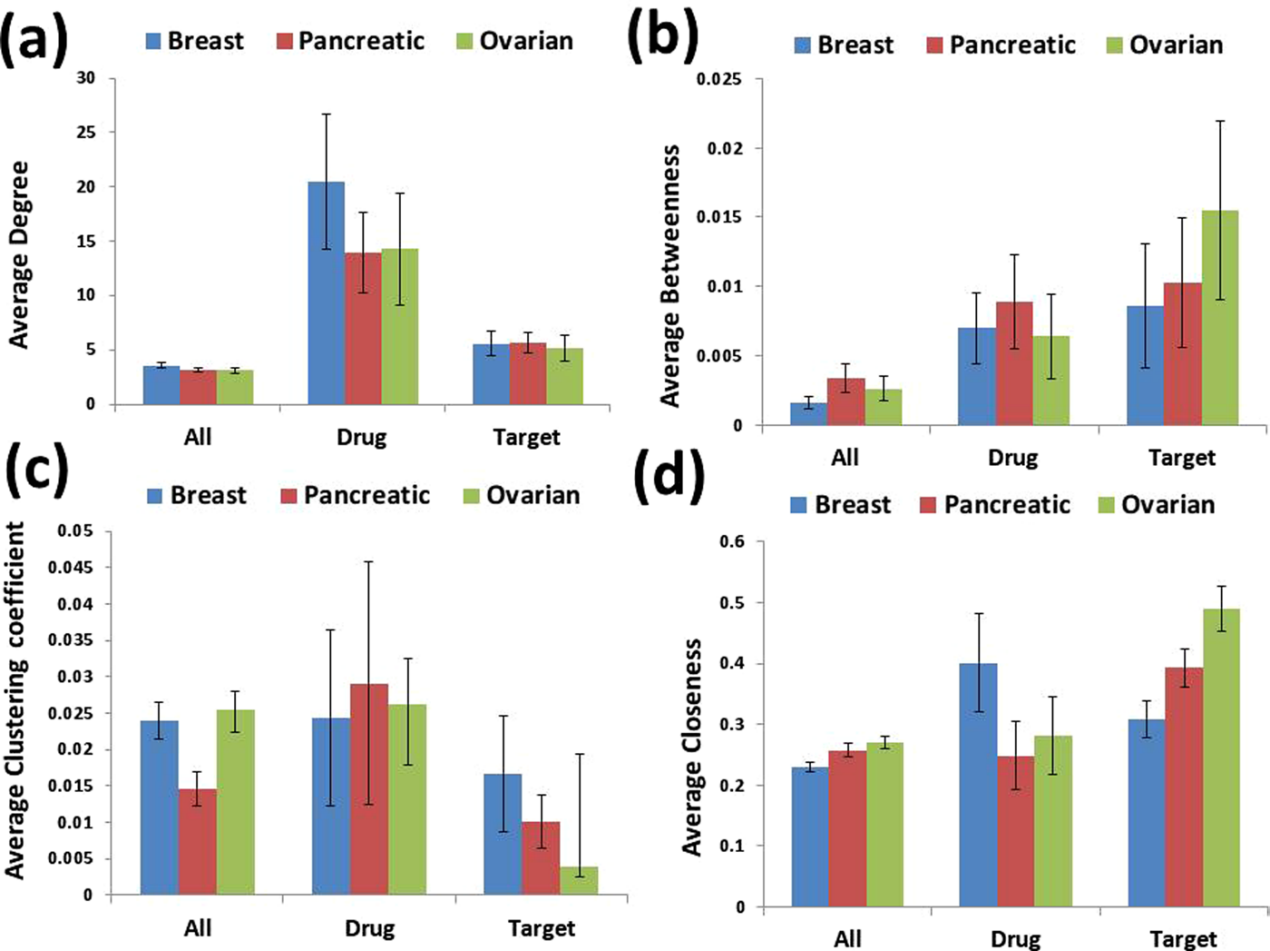
Topological properties of drug-target and target (essential) proteins in compare to whole network. (**a**) Average degree of drug-target and target proteins in compare to whole network. (**b**) Average betweenness of drug-target and target proteins in compare to whole network. (**c**) Average clustering coefficient of drug-target and target proteins in compare to whole network. (**d**) Average closeness of drug-target and target proteins in compare to whole network.

We analyzed several topological properties of the drug target proteins included by our algorithm in the set of driver nodes, and of the essential proteins in each of the networks in our study. We looked at the average degree, the betweenness centrality, the closeness centrality, and the clustering coefficient of these proteins as compared with the average values over the entire networks. We found that in all the three considered cancer networks, the drug-target driver nodes and the essential proteins have much higher average degree than the average over the whole networks, Fig. 3. This shows that both the drug-target driver nodes and the essential proteins are hubs in the networks and thus central in the regulation of the networks; this is consistent with observation of, e.g. refs 31, 32. The essential proteins were found to have a higher average betweenness centrality than the average over the whole networks, especially in the breast and in the pancreatic cancer networks, Fig. 3. This indicates that essential proteins act as highly-traversed bridges in these interaction networks; nodes with high betweenness centrality values have been reported also in several other pathways, including MAPK pathways^33, 34^.

The other topological indicates we considered did not systematically distinguish the drug-target driver nodes or the essential proteins against the rest of the networks nodes, Fig. 3.

### From driver nodes to combinatorial drug therapy strategies

**Figure 4:**
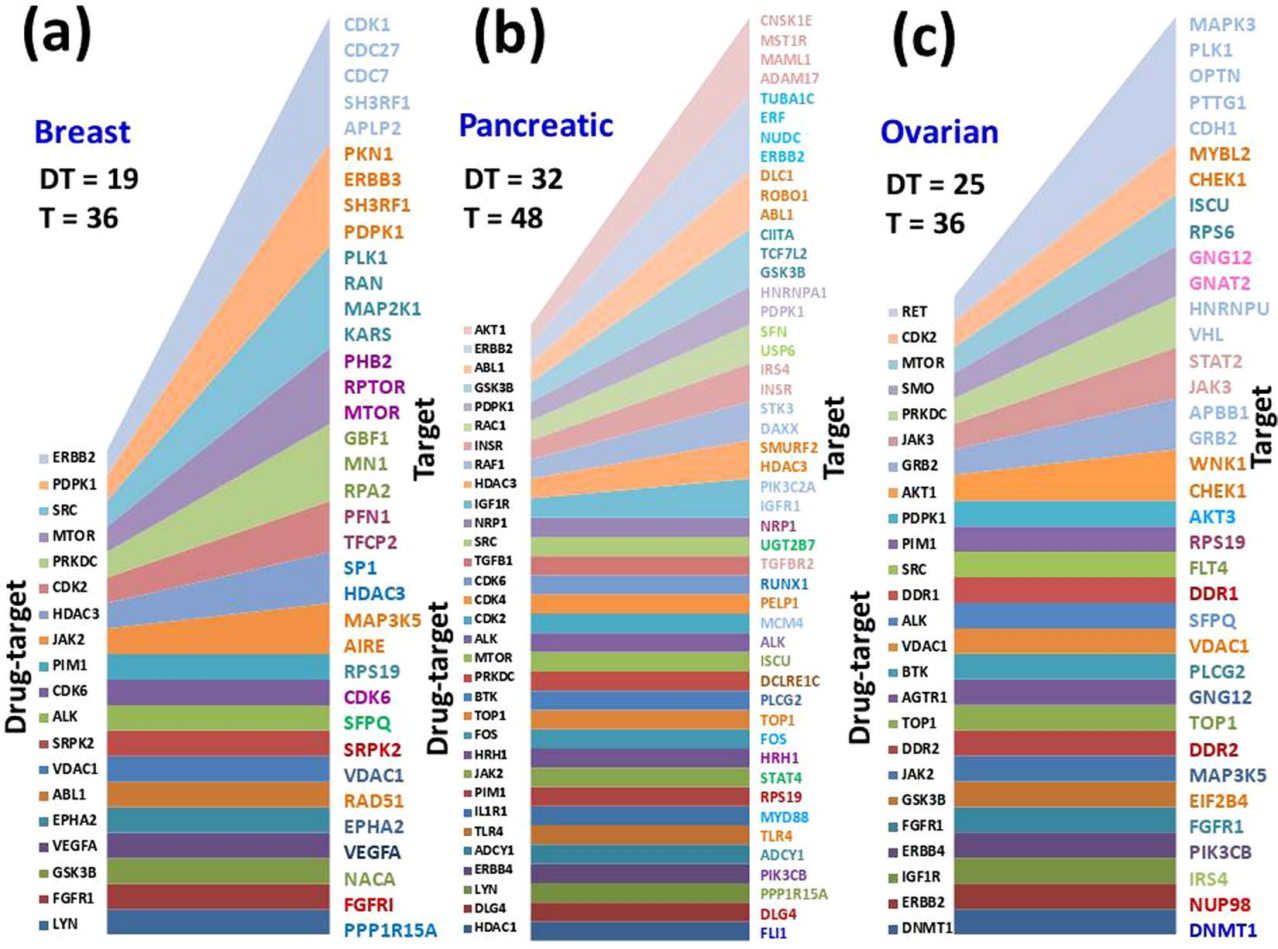
Target control efficiency of drug-target proteins. (**a**) Control features of drug-target proteins in breast cancer. (**b**) Control features of drug-target proteins in pancreatic cancer. (**c**) Control features of drug-target proteins in ovarian cancer.

We analyzed the drug-targetable proteins identified by our algorithms as part of the strategies to control the cancer essential proteins. We found that some of them are themselves oncoproteins and thus could be a direct target in cancer therapy. Among those that are not oncoproteins, we found that some have a high impact in their corresponding network, controlling several essential proteins simultaneously. One of them is ERBB2, which controls five essential proteins in breast cancer (CDK1, CDC27, CDC7, SH3RF1, APLP2) and four essential proteins in pancreatic cancer (CNSK1E, MST1R, MAML1, ADAM17); Fig. 4 and Table 4. This is in line with previous observation of ref. 35 showing that ERBB2 is often a drug-target in cancer therapies. Another potent drug-target protein is RET, controlling five oncoproteins (MAPK3, PLK1, OPTN, PTTG1, CDH1) in the ovarian cancer network, Fig. 4 and Table 4. The list of all high impact drug-target protein (controlling more than two essential proteins) is in Table 4. We observed that out of the 75 drug-target proteins included by our algorithms in the control strategies (driver nodes) of the three cancer networks, 31 of them are present in more than one cancer network. This shows that they are expressed in multiple cancer cell lines and could be used in drug therapies of several cancers, in combinations with cancer type-specific targets.

**Table 4:** Highly impact drug-target proteins for Breast, Pancreatic and Ovarian cancers.

Cancer Types	Drug-target	Target proteins	Anti-cancer drug	Known to be used in cancer therapies
Breast	ERBB2	CDK1, CDCH2, CDC7, SH3RF1, APLP2	Lapatinib	Breast, Lung
	SRC	PLK1, RAN, MAP2K1, KARS	Dasatinib, Bosutinib, Ponatinib	Chronic myelogenous leukemia (CML)
	PDPK1	PNK1, ERBB3, SH3RF1, PDPK1	None	None
	PRKDC	GBF1, MN1, RPA2	None	None
	MTOR	PHB2, RPTOR, MTOR	Temsirolimus	Renal cell carcinoma (RCC), Bone marrow cancer,
	JAK2	MAP3K5, AIRE	Ruxolitinib, Erlotinib	Pancreatic cancer and others types of cancer
	HDAC3	SP1, HDAC3	Vorinostat	Cutaneous T cell lymphoma (CTCL)
	CDK2	PFN1, TFCP2	None	None
Pancreatic	ERBB2	TUBA1C, ERF, NUDC, ERBB2	Lapatinib	Breast, Lung
	AKT1	CNSK1E, MST1R, MAML1, ADAM17	None	None
	GSK3B	DLC1, ROBO1, ABL1	Hepatitis B immune globulin, Alectinib, Paclitaxel, Eribulin, Testolactone	Liver cancer, Anaplastic lymphoma kinase (ALK) and Non-small cell lung cancer (NSCLC), Cancer chemotherapy, Breast cancer
	ABL1	DLC1, ROBO1, ABL1	None	None
	IGF1R	PIK3C2A, IGFR1	None	None
	HDAC3	SMURF2, HDAC3	Vorinostat	Cutaneous T cell lymphoma (CTCL)
	RAF1	STK3, DAXX	None	None
	INSR	IRS4, INSR	None	None
	RAC1	SFN, USP6	None	None
	PDPK1	HNRNPA1, PDPK1	None	None
Ovarian	RET	MAPK3, PLK1, OPTN, PTTG1, CDH1	Cabozantinib, Lenvatinib, Vandetanib, Sunitinib, Regorafenib, Ponatinib,	Medullary thyroid cancer (MTC), Thyroid cancer, Renal cell carcinoma (RCC), Imatinib-resistant gastrointestinal stromal tumor (GIST), Metastatic colorectal cancer and Advanced gastrointestinal stromal tumours, Chronic myeloid leukemia,
	AKT1	WNK1, CHEK1	None	None
	GRB2	APBB1, GRB2	None	None
	JAK3	STAT2, JAK3	None	None
	PRKDC	HNRNPU, VHL	None	None
	SMO	GNG12, GNAT2	Vismodegib, Sonidegib	Basal cell carcinoma
	MTOR	ISCU, RPS6	Temsirolimus	Renal cell carcinoma (RCC)
	CDK2	MYBL2, CHEK1	None	None

The columns represent the type of cancer, drug-target, target (essential) proteins, name of anti-cancer drug, and type of cancer for which the drug is known to be used.

We looked for anti-cancer drugs for the drug target proteins identified by our control algorithms. We found that in some cases they are used in current cancer type-specific drugs and drug-therapies; for example, anti-cancer drugs targeting the ERBB2 gene are in use for breast cancer. In many other cases however, we found that the drug-targets identified by our methods are either not used in any known cancer therapies, or at least not in the case of the specific cancers we analyzed. These results and observations are summarized in Table 4.

### Functional properties of the high-control proteins

We reviewed the functional properties of the driver nodes found to have the highest impact in controlling the essential proteins; they are ERBB2, SRC, PDPK1, PRKDC, mTOR for breast cancer, ERBB2, AKT1, GSK3B, ABL1 in pancreatic cancer, and RET in ovarian cancer. Our goal was to correlate the findings of our computational study with previous studies on the functional properties of these proteins.

In breast cancer, the ERBB2 oncogene activates signaling pathways that deregulate the essential protein processes and make cancer cells resistant to chemotherapeutic drugs of cancer cells^36^. Amplification of ERBB2 gene is the main cause of its over-expression in cancer^37^, while depletion of glucose also inhibits expression of ERBB2^36^. Moreover, the PI3K/AKT pathway directly activates the mutations in ERBB2-amplified breast cancers^38^. The SRC protein is activated by various factors such as cytoplasmic proteins, which play vital role in integrating signalling and ligand activation of cell surface receptors. These interactions interrupt the intermolecular interaction within SRC and lead to over-expression of upstream growth factor receptors^39^. Other intrinsic factors in breast cancer are dephosphorlization of SRC, SRC regulation by RTKs, and SRC activity gene expression signature^40^.

The protein PDPK1 has a crucial role in the over-proliferation of breast cancer^41^, while^42^ shows that anchorage-independent growth is regulated by PDPK1, which resists to many anti-cancer drugs and starts the tumour formation in breast cancer cell lines. Along with this, PDPK1 proteins phosphorylate the activating segment of AKT, which affects various key cell functions and facilitate the breast cancer progression^41^. The PRKDC protein is also carrying an important role in breast cancer^43, 44^. Downregulation of MYC mRNA and protein expression in multiple cancer cell lines is caused by inhibition of PRKDC, which leads to over-expression of MYC family of proteins induced DNA double-strand breaks and leads to cancer progression^45^. Protein mTOR, together with PIKS and Akt, mediates multiple cellular pathway functions. Aberrations and degradations inside these pathways leads to tumour proliferation in breast cancer^43, 46^. These aberrations affect germline and somatic mutations, amplification, rearrangements, methylation, overexpression, aberrant splicing, and starts mutation in breast cancer cell lines^47^.

In pancreatic cell lines, overexpression of ERBB2 is known to advance the disease states^47^. Moreover, knocking down of CAPAN-1 and CAPAN-2 cells by ERBB2 increases the sensitivity to gemcitabine, the resistance to irinotecan/SN-38, the increase of hCNT1 and hCNT3 transporters, and ABCG2, MRP1 and MRP2 ATP-binding cassette transporters expression, which leads to apoptosis^48^. *In vivo*, PEAK1-dependent kindles induced by oncogenic KRas amplify the loop between SRC, PEAK1, and ERBB2 drive pancreatic cancer. Also, increased SRC-dependent PEAK1 expression by blockade of ERBB2 expression activates tumour growth^49^. The next protein in our list is AKT1, which is serine/threonine kinase AKT (also known as Protein Kinase B) for which we found reports of over-expression in pancreatic tumour formation^50^. The alteration of AKT increases the oncogenic changes in tumour, and the activation of AKT isoforms disturbs the down-regulation of pancreatic tumours which starts upstream signalling^50^. Also, the activation of HER2/3- PI3K/Akt signaling pathways by VIP plays a key role in growth and survival of cancer^51^. For the next protein in our list GSK3B, we found reports that its inhibition activates JNK-cJUN-dependent apoptosis in human pancreatic cancer cell lines^52^ and participates in the nuclear *factor*–*kβ* (*NF*–*kβ*) mediated cell survival in pancreatic cancer^53^. Also, GSK3B is documented to initiate the tumour through activation of the oncogenic *β*-catenin^54^, which over-expressed the GSK3B in pancreatic cancer. The next protein ABL1 is over-expressed in pancreatic cancer^55^. Alteration in ABL mRNA expression in tumours increases the activity of ABL kinase, which promotes the cancerous’ cell over-proliferation and survival^56^. Interestingly, cellular stress and DNA damage induced ABL1 escalate the cell growth arrests or apoptosis mediated by p53 or p73^57^.

In ovarian cancer RET (REarranged during transfection) is expressed and involved in pathogenesis of ovarian cancer^58, 59^. RET tyrosine kinase is a fusion partner of TRIM27 (tripartite motif-containing 27), which is highly expressed in normal epithelial cells of the ovary and fallopian tube and in ovarian serous carcinoma cells^60^. It has been pathologically characterized in patients with ovarian serous carcinoma. Since RET participates in essential cellular processes, the over-expression of fusion proteins (TRIM27-RET) disrupts its essential cellular activity and triggers tumorigenesis^60^.

## Discussion

We analyzed the breast, pancreatic, and ovarian cancer protein-protein interaction networks, and identified the respective sets of driver proteins for controlling the networks. Recent genetic editing technologies explain the existence of cancer-specific sets of proteins which have an important role in the overall disease mechanism. These proteins, called cancer survivability-essential proteins, are proved to be key for *in-vivo* cancerous cell’s proliferation and survival. Therefore, instead of trying to achieve a full control of the entire disease’s network, which in itself is highly complex, our approach aims for a targeted control approach, particularly for controlling those cancer essential proteins. In order to achieve the partial control of all the above mentioned cancers, we have first generated for each of them the associated signal transduction directed protein-protein interaction network. These networks identify the in-between influence of the proteins passed on their overall expression levels. Our analysis showed that in order to control all of the essential proteins in these cancer networks, we require the direct intervention over only 6.6–13% of the entire networks’ nodes, Table 2. In turn, to achieve a full control of these networks, it required around 70% of the networks’ nodes to be directly controlled by an outside intervention, Table 1, e.g., such as achieved by administering a number of drugs. Thus, our method generates up to a 10-fold decrease in the control effort, while maintaining a high likelihood of an overall similar effect. Moreover, our methodology and algorithms for target control of the essential proteins emphasize, and maximize, the use of known drug target proteins, as a choice for input controlling nodes, i.e., driver nodes, of the network.

Furthermore, we analyzed the topological properties of the driver DT proteins and of the essential proteins in all cancer networks, in order to understand the structural and functional properties of these proteins. We observed that driver DT-associated nodes have high degrees in the network, see Fig. 3, which shows that these proteins are central, and that they form robust connections inside the networks. This characteristic seems to confirm the control efficiency of these nodes, as it shows that these proteins have multiple connections within the networks and this intensifies the feasible control over the target (essential) nodes. Also, we observed that the essential proteins have high betweenness centrality, Fig. 3, showing that these proteins operate as a bridge in the networks, and that they are highly important for the signal flow.

To make our approach practical it is important to construct cancer type-specific network models. We started the construction of our models from cancer-specific data from the UniprotKB protein database, as well as cell line-specific survivability-essential genes from the COLT-Cancer database^29^ and extracted the directed PPI network connecting them from the SIGNOR database. This method can be enriched with additional data, such as cancer hallmark data, including cell survival, mutation, and epithelial-mesenchymal transition networks, as demonstrated in ref. 61 and discussed more generally in ref. 62. The general goal of integrating multiple data sources is to make the network model (and thus, the results of our methods) as specific as possible. It is also important to address the diversity of the tumor subclonal networks, see ref. 63. Our approach seems promising here: given the large number of combinatorial predictions generated for a given network, it is possible that the predictions for subclone-specific networks could overlap and thus offer a simultaneous solution to several tumor subclones. Another possibility is to enrich the set of driver nodes controling a clone with driver nodes needed to control another clone; our results in Supplementary Note S9: show that the enriched set will then control the essential genes of both clone networks, albeit through a potentially larger set of drug-targets.

We analyzed the relationship between the driver DT proteins predicted by our algorithm and cancer therapies. We observed that some of these DT proteins are oncoproteins, and thus the associated targeting drugs have a strong potential therapeutic effect in those cancers, Table 4. Other driver DT proteins based drugs are known to be part of therapies in other cancers, but not in breast, pancreatic or ovarian. We also observed that out of all selected 75 driver DT proteins in all three cancer networks, 31 DT proteins are present in more than one cancer. This shows that some DT proteins are expressed in multiple cancer cell lines. We also analyzed the functional properties of high control proteins in all cancers, and found that these proteins are directly responsible for the occurrence of particular cancers.

The control methodology applied in this study provides an efficient way to control an interactome network through known drug target nodes, especially in the case of disease associated networks. Also, this work provides a better understanding of the disease associated biochemical networks and opens a new way towards the successful application of drug-target based control mechanisms. This in turn could pave the way for future studies of various disease diagnostic techniques based on network controllability, efficient therapeutic approaches, and personalized medicine.

## Materials and Methods

### Cancer data

The cancer data used in this study was obtained both from the publicly available UniprotKB protein database^64^, as well as from the literature^28, 65–71^. We concentrated our study over three types of cancer, namely breast, pancreatic and ovarian, for which we gathered data for 1415, 991 and 1047 proteins respectively, see Table S1. We used short Python scripts to check for redundancy in the gathered data.

### Protein-protein interaction data

To obtained directed PPI (signalling) cancer data, we used SIGNOR (SIGnaling Network Open Resource) database^72^, which outputs binary matrix representations for the user-provided protein lists; this allowed us to create directed graphs between signaling entities. We obtained directed PPIs networks of 2532 interactions from 1415 nodes in breast cancer, 1569 interactions from 991 nodes in pancreatic caner, and 1643 interactions from 1047 nodes in ovarian cancer. The networks are available for download at ref. 73, as well as in Table S2.

### Essential protein data

Although diseased cells may harbor hundreds of genomic alterations in various biological pathways^10, 24^, only a subset of these alterations are driving the disease initiation and progression. These proteins form together the sets of (disease-specific) essential proteins. Due to the new CRISPR gene editing technology, researchers can now pinpoint essential proteins for a very large class of illnesses^21^, including many types of cancers^20, 74^. We collected essential gene data for breast, pancreatic, and ovarian cancer from the COLT-Cancer database^29^. In particular, we considered the MDA-MBD-231, HPAF-II and OV-90 cell lines respectively for breast, pancreatic and ovarian cancer, and follow the GARP (Gene Activity Rank Profile) and GARP-P value of corresponding proteins mentioned in the database. Since previous studies showed that proteins with lower GARP score are more essential and directly associated with oncogenesis^74^, we selected only those essential proteins whose GARP value is in the negative range, and moreover, whose GARP-P value is less than 0.05 (p ≤ 0.05). Following the above criteria, we identified 712, 770 and 866 proteins respectively for breast, pancreatic and ovarian cancer, see see Table S3. Out of these, 135, 168 and 140 essential proteins respectively in breast, pancreatic and ovarian cancer were found available in the SIGNOR PPI network database, and were included in our network.

### Drug target data

We obtained drug-target protein data from the open source DrugBank database^75^. The DrugBank database offers extensive information of drug and drug targets. This includes information of chemical, pharmacological and pharmaceutical specific drugs integrated with structure, pathway and sequence drug target. For drug-target identifiers we have selected in total 1507 FDA-approved proteins which have a known mechanism, see Table S4.

### Theoretical model and optimization algorithm

The mathematical methodology used for deriving the sets of proteins through which we can effectively manipulate the system, aka driver nodes/proteins (in some literature, these nodes are also called driven or controlled nodes, while the driver nodes are some outside system actuators directly influencing the network’s driven nodes.), is based on the well established Structural Control Theory. This theory, although thoroughly investigated since the 70’s, e.g. in the works of Kalman^76^, Lin^77^, Murota^78^, etc., has recently received a new boost of attention^23, 25, 79, 80^, partly due to recent results on efficient algorithms for core research problems within this framework.

We say that a dynamical system, such as the expression levels of a set of genes/proteins influencing each other, is *controllable* from a set of input (*driver*) nodes, if there exists a time-dependent sequence of input signals delivered through these nodes such that the system can be driven from any initial state to any desired final state within finite time. From the point of view of our study, we can concentrate over linear time-invariant dynamical systems (LTIS). Such systems can be visualized as directed networks, where the nodes represent the components of the system while the weighted directed edges represent how these components interact and influence each other. A more mathematically rigourous definition of LTIS and of previous results is presented in Supplementary Note S7: In a recent breakthrough an efficient (low polynomial time) algorithm was provided for computing the minimal number of input nodes needed to structurally control any given LTIS network^25^. However, it was also shown that in the case of sparse inhomogeneous networks, such as most of the networks emerging from biochemical and biomedical applications, controlling the entire system is expensive, requiring up to 80% of the system’s nodes to be controlled directly. On the other hand, in terms of practical applications, in many cases it is enough to control only a certain well-selected portion of the network’s nodes, such as the set of essential proteins, in order to impose a certain overall behaviour over the system. Thus, controlling those target proteins, or a considerable subset of them, could translate into a highly effective control approach over the desired system dynamics.

Our algorithms, see Supplementary Note S8: aim to minimize the number of driver nodes (i.e., network nodes) which can be used to control a given target, namely the set of cancer-specific essential genes in each network. Our approach is different than in ref. 23 that minimizes the number of outside input nodes (i.e., possibly acting upon several of the network nodes in the same time). The rationale for this choice is that we aim for combinatorial drug target identification and we consider only the primary target of each drug under consideration. Our algorithm has a double optimization objective, namely to minimize the total number of driver nodes and to maximize the percentage of FDA-approved target nodes among them. We used several heuristic strategies for a more efficient exploration of the search space, aiming for faster algorithms and better optimizations.

We implemented an additional validation step for the proposed solution of our algorithm, which is freely available at ref. 73. An example in ref. 78 shows that in some rare cases, the algorithm in ref. 23, whose basic search strategy we also follow here, may output a non-solution (a set of nodes that fails to control the given target). In such a case, we restart the search algorithm and given the built-in randomness of our algorithm, we expect to get another candidate solution with high probability. The size of the set of non-solutions outputted is not-known in general but according to ref. 71, it is expected to be very small.

### Topological properties of networks

The *degree* of a node in a network is the number of connections the node has to other nodes. The robustness of a network depends upon the connections between the nodes inside the network. Another important node-associated value is the *cluster coefficient*, which, for a node *v*, is defined as ${C}_{v}=n/{k}_{v}({k}_{v}-\mathrm{1)}$, where *k*_*v*_ is the number of neighbours of *v* and *n* is the total number of connections/edges between these neighbours. The clustering coefficient of a node takes values between 0 and 1, where 1 implies that the node *v* is in a complete sub-graph, while 0 denotes that the node is part of a loosely connected cluster (a star-shaped cluster with v in the centre). Further, the *betweenness centrality* of a node *v* is defined as the weighted sum of all shortest pathes between all pairs of nodes *s* and *t*, that go through the node *v*. That is, ${C}_{B}(v)={\sum }_{s\ne v\ne t}\frac{{\sigma }_{st}(v)}{{\sigma }_{st}}$, where *σ*_*st*_ is the number of shortest paths between nodes *s* and *t*, while *σ*_*st*_(*v*) is the number of such shortest paths running through node *v*. Also, the *closeness centrality* of a node indicates how close this node is from all other nodes; it is defined formally as ${C}_{c}(v)={\sum }_{t\in V\backslash \{v\}}\frac{S(s,t)}{n-1}$, where *S*(*v*, *t*) is the shortest path between *v* and *t*.

### Data availability

All data generated or analysed during this study are included in this published article (and its Supplementary Information files).

## Electronic supplementary material

Supplementary File

Table S1

Table S2

Table S3

Table S4
