# Supplementary material for: Controlling Directed Protein Interaction Networks in Cancer
Source: Sci Rep. 2017 Sep 4;7:10327. doi: 10.1038/s41598-017-10491-y (PMC5583175; doi:10.1038/s41598-017-10491-y)
Supplement: Supplementary file 1 — Supplementary File [file 41598_2017_10491_MOESM1_ESM.pdf]

Supplementary Information of Controlling Directed Protein Interaction Networks in Cancer

Krishna Kanhaiya[1], Eugen Czeizler[1], Cristian Gratie[1], and Ion Petre[1,*]

[1]Computational Biomodeling Laboratory, Turku Centre for Computer Science, and Department of Computer Science, ˚Abo Akademi University, Turku, 20500, Finland
[*]ipetre@abo.fi

ABSTRACT

Control theory is a well-established approach in network science, with applications in bio-medicine and cancer research. We build on recent results for structural controllability of directed networks, which identifies a set of driver nodes able to control an a-priori defined part of the network. We develop a novel and efficient approach for the (targeted) structural controllability of cancer networks and demonstrate it for the analysis of breast, pancreatic, and ovarian cancer. We build in each case a protein-protein interaction network and focus on the survivability-essential proteins specific to each cancer type. We show that these essential proteins are efficiently controllable from a relatively small computable set of driver nodes. Moreover, we adjust the method to find the driver nodes among FDA-approved drug-target nodes. We find that, while many of the drugs acting on the driver nodes are part of known cancer therapies, some of them are not used for the cancer types analyzed here; some drug-target driver nodes identified by our algorithms are not known to be used in any cancer therapy. Overall we show that a better understanding of the control dynamics of cancer through computational modelling can pave the way for new efficient therapeutic approaches and personalized medicine.

Supplementary Note S7: Structural control theory, definitions and known results

Given an n-dimensional vector of variables $x(t) = (x_1(t), ..., x_n(t))^T$ and a state transition matrix $A \in R^{n\times n}$ we define a *linear, time invariant dynamical system* (LTIS) as

$$\frac{dx(t)}{dt} = Ax(t). \tag{1}$$

If the system is (linearly) influenced by a size-m external input controller $u(t) = (u_1(t), ..., u_m(t))^T$, where $B \in R^{n\times m}$ (i.e., the *input matrix*) describes how the n variables are affected by the m inputs, then system (1) becomes:

$$\frac{dx(t)}{dt} = Ax(t) + Bu(t).$$

In the additional case when the system is also exporting a set of k output values, $y(t) = (y_1(t), ..., y_k(t))^T$ depending on the current state $x(t)$, the system (1) becomes:

$$\begin{aligned}\frac{dx(t)}{dt} &= \quad Ax(t)\\ y(t) &= \quad Cx(t)\end{aligned}$$

where $C \in R^{k\times n}$ is the *output matrix*. In the particular case when the output consists of a k subset $T \subseteq X$ of the total n variables, such as a target set, the output matrix C_T is a 0-1 matrix, with $C_T(i, j) = 1$ iff $i = j$ and $i, j \in T$. We will denote by (A,B,C) an m-input, k-output LTIS, where $A \in R^{n\times n}$, $B \in R^{n\times m}$, and $C \in R^{k\times n}$.

Given a target set T, an LTIS (A,B,C_T) is said to be *target controllable* if there exists an input vector $u(t) = (u_1(t), ..., u_m(t))^T$ that can drive the state of the target variables to any desired numerical setup in finite time. A system (A,B,C_T) is target controllable if and only if (see[1]):

$$\text{rank}[C_TB, C_TAB, , C_TA^2B, ..., C_TA^{n-1}B] = |T| \tag{2}$$

An LTIS (A,B,C_T) is *structurally target controllable* if there exists a time-dependent input vector $u(t) = (u_1(t),...,u_m(t))^T$ and a numerical setup for the non-zero values within the matrices A and B, that can drive the state of the target nodes to any desired numerical setup in finite time. That is, (A,B,C_T) is structurally target controllable if and only if there exist values for the non-zero entries in A and B such that

$$\text{rank}[C_T B, C_T AB, , C_T A^2 B, ..., C_T A^{n-1} B] = |T|.$$

It is known, see e.g.[2,3], that if a system is structurally (target) controllable, then it is (target) controllable in almost all numerical setups of the non-zero values within the state transition matrix A.

We will equivalent (structural) LTIS with directed (weighted) networks. The n variables of the systems are the nodes of the network, while directed edges correspond to non-zero values in the state transition matrix. Similarly, the size-m controller vector u corresponds to m input nodes, $u_1,...u_m$, also called *driver nodes*, while the input matrix B determines the edges between the driver nodes and the network, i.e., (u_i,x_j) form a directed edge, with weight w, if and only if $B(x_j,u_i) = w \neq 0$. The nodes x_j such that there exists i with $B(x_j,u_i) \neq 0$ are called the *driven nodes* of the network; these are the first nodes in the network which are directly manipulated in order to drive the entire system to the desired state. It is known, see e.g.[2], that the target structural controllability problem has a counterpart formulation in terms of networks. The LTIS (A,B,C_T), where $|T| = k \geq 1$, is structurally target controllable from the m-input/driver controller u and control matrix B if and only if we can select a set of k directed paths from the input/driver nodes (i.e., as starting points) to each of the target nodes (i.e., as ending points), such that no two paths would intersect at the same distance d from their end points.

From the point of view of bio-medical disease network analysis and control, it is sometimes more advantageous to consider the set of driven nodes instead of that of the driver nodes. To a rough understanding, the set of driver nodes is describing the complexity of an outside controller, assuming this controller can interact/influence several of the network nodes; such an interaction could be seen for example as the influence of a drug over the expression of some particular genes. Meanwhile, the set of driven nodes provide the exact collection of network nodes, i.e., genes, that will be used in order to ultimately control the entire set of target nodes. In particular, if we require that each driver node is interacting with at most one network node (as it might be the case of certain drugs), then the control matrix B has at most one non-zero entry for every column, and there is a one-to-one correspondence between driver and driven nodes. Thus, in this study, we concentrate over minimizing the set of driven nodes for the control of a given target within a directed network.

In[4] the authors prove that the problem of selecting a minimal set of driven nodes which would control a given target within a directed network is algorithmically hard, i.e., NP-hard. In the same time, the authors introduce a heuristic approximation algorithm which they use successfully on networks of up to 3000 nodes and 10000 edges.

Supplementary Note S8: Structural target control with preferential operators

The output of the above target control approaches is a (close to minimal) set of nodes which can be used to control a given target within a network. However, no information of the availability of directly influencing these chosen nodes is taken into consideration when selecting the controlling set. In practice however, some nodes from these networks would be much prone to direct influencing than others. In case of bio-medical networks for example, when the nodes represent various proteins or genes, such preferential choices are those nodes (i.e., proteins, genes) which are drug-targets of known (and/or FDA-approved) drugs. Thus, out of the entire network that is given, we could select a list of endorsed nodes from which we would want to either control the entire target, or, maximize the selection of these preferred nodes as driven nodes, while still following the objective of selecting a close to minimal set of driven nodes, i.e., a type of *min-max algorithmic optimization problem*. We will call such a list of endorsed nodes as the preferred list of operators.

It is note observing that deciding whether a given target can be controlled from a preferred list of operators $O \subseteq V$ is a relatively easy tasks, equivalent to computing the rank of the structural matrix 2 for the case when B is the structural matrix B_O (where the matrix B_O is obtained in the same way as the output matrix C_T for a target T).

In[4], an NP-hardness proof was provided for the structural target control problem. Although it was not intended, the construction therein can be used as is for proving that also the structural target control with preferential operators problem is also NP-hard; one has to define the preferred list of operators O as containing all *"valuation nodes"*, as defined therein.

Theorem: The structural target control with preferential operators problem is NP-hard.

The idea of the NP-hardness proof from[4] is via an embedding of the problem to 3SAT. Given a 3SAT formula P over n boolean variables and m clauses, the authors construct a directed network, involving also $2n$ valuation nodes and m clause nodes with the following property. Assuming the target T consist of all the m clause nodes, the authors prove that the formula P is satisfiable if and only if the target T can be controlled from exactly n driven nodes, and, moreover, at most one of the

valuation nodes associated to a boolean variable can be selected in this pool of control nodes. Thus, by selecting the preferred list of operators O as containing all the $2n$ valuation nodes we see that the problem of minimizing the size of the control set while maximizing the use of preferred operators is also NP-hard.

Building on the approximation algorithm from[4] for structural target controllability we introduce here a new algorithm for structural target control with preferential operators. Namely, given a directed network (e.g., a protein/gene signalling network), a set of target nodes (e.g., a set of disease-specific essential genes), and a set of preferential operators (e.g. a set of genes/proteing known to be directly targeted by specific drugs) all within the network, find a close to minimal set of nodes that maximizes the use of the available operators, in order to control the targets. We detail bellow this algorithm.
Note: Given two sets A and B, we denote by $A \cup B$ and $A \sqcup B$ the union and disjoint union, resp., of these sets.

Let $G = (V, E)$ be a directed graph, let $T \subseteq V$ be the set of target nodes, and let O be a set of preferential operator nodes. We construct a sequence of sets $C^i, D^i, i \geq 0$, (and $i \leq |V|$) with $C^0 = T$, $D^0 = \emptyset$, and $|C^i| \geq |C^{i+1}|$, such that the union set $\bigcup_{1 \leq k \leq i} D^k$ is a set of nodes controlling the target T, where the use of the nodes in O is maximized in the generation of D; we refer to[4] for the explanation on why this claim holds.

The $TarCoOp$ algorithm for target structural control with preferred operators:

Step 1 i:=0, $C^0 := T$, $D := D^0 := \emptyset$

Step 2 Define the bipartite graph $G^i = (L^i \sqcup R^i, E^i)$, where $L^i = V, R^i = C^i$, and E^i contains edges $(l, r) \in (L^i, R^i)$ such that $(l, r) \in E$ is an edge also in the initial graph.

Step 3 Find a maximum (cardinality) matching (M^i_L, M^i_R) (following the 8 heuristic criteria below) in G^i, where $M^i_L \subseteq L^i$ and $M^i_R \subseteq R^i$, and let $C^{i+1} = M^i_L$ be the set of the left sided matched nodes and $D_i = R^i \setminus M^i_R$ be the set of right sided un-matched nodes.

Step 4 For each $x \in D_i \setminus D$

Step 5 If $x \in \bigcup_{j<i} C^j$ (i.e., x appears in any previously computed C^j, $j < i$)

Step 5.1 remove the entire control path from that occurrence (in C^j) onward, and update all the sets C^k, D^k with $j \leq k \leq i+1$ accordingly.

Step 5.2 Update D as $D := \bigcup_{0 \leq p < i} D^p$.

End If (from Step 5)

End For (from Step 4)

Step 6 Update D as $D := D \cup D^i$ and C^{i+1} as $C^{i+1} := C^{i+1} \setminus D$.

Step 7 If $C^{i+1} = \emptyset$ then output D as a set of control nodes for T and stop. Else proceed to Step 8.

Step 8 If $i < n$ then $i := i+1$ and proceed to Step 2. Else, proceed to Step 9.

Step 9 For all the remaining nodes in C^n, add them one by one to the driven set D and, at each new addition to D, perform the check from Step 5, i.e., pruning the existing controlling path for each new addition in D.

Step 10 Output D as a set of control nodes for T.

On Step 3 above we mention 8 heuristic criteria for implementing a maximum (cardinality) matching in between the left, $L^i = V$, and right, $R^i = C^i$, disjoint sets of the bipartite graph G^i. This is due to the fact that the maximum matching might not be unique, and, depending on which maximum matching we chose, the size of the final set controlling the target nodes can differ significantly. Also at this point in the algorithm we can intervene so that a maximal amount of preferred operators is chosen as actual driven nodes. In the following we are introducing this set of 8 heuristic criteria.

- Criteria 1: All preferred nodes from O appearing in a control path are directly controlled, i.e., the maximum matching is performed between sets $L^i := V$ and $R^i := C^i \setminus O)$ while $D^i := D^i \cup \left(C^i \cap O\right)$,
- Criteria 2: Maximize the use of already driven nodes in M^i_L.

- Criteria 3: Maximize the use of preferred operators in M_L^i. This is done by initiating the maximum matching algorithm by a first (maximal) matching which maximizes the number of pairs $(x,y) \in (M_L^i, M_R^i)$ where x is a preferred operator,i.e., $x \in O$.
- Criteria 4: Try to avoid the creation of cyclic controlling path. That is, avoid selecting nodes $x \in M_L^i$ such that there exists $j \leq i$ and a sequence $u_{i+1},...,u_j$ such that $u_k \in C^k$ for all $j \leq k \leq i$, $u_{i+1} = u_j = x$, and for all $j \leq k \leq i$, u_j is matched to u_{j+1} in the corresponding bipartite graph.
- Criteria 5: Maximize the use of nodes in M_L^i which have appeared in some previous $C^j, j < i$, on a path that is already controlled (ends with a driven node).
- Criteria 6: Maximize the use of nodes in M_L^i which have appeared in some previous $C^j, j < i$, on a path that is not controlled yet.
- Criteria 7: Maximize the use of edges (u,v) (with $u \in M_L^i$ and $v \in M_R^i$) which have been used in some previous matching and are part of at least one path that is already controlled.
- Criteria 8: Maximize the use of edges (u,v) (with $u \in M_L^i$ and $v \in M_R^i$) which have been used in some previous matching, but are not part of any path that is already controlled.

In performing the TarCoOp algorithm, we can choose to follow the above criteria either in the order of importance Cr1 - Cr8, or in the order Cr1-Cr4, Cr7, Cr8, Cr5, Cr6, in which case we obtain a slightly different outcome. In practice, which of these two versions of the algorithm performs best is a feature strongly depending on the intrinsic properties of the considered network. Thus, in our implementation, we run both algorithms repeatedly (sequentially or in parallel), either for a fixed amount of time , e.g., 12 hours, or until the optimum solution does not get updated after 50 runs.

Supplementary Note S9: Robustness to Network's False Negative interactions

Many times a given bio-medical network reflects not the complete truth, but only the currently available information. While careful curation of data ensures a low level of false positives in the network, i.e., all the provided signalling and/or interaction edges are carefully validated, false negatives, i.e., no edge is present in the network while one should be there, are instances of no research being yet performed regarding such connections. However, as research on the topic progresses, such new edges might in time become available. The question regarding our target network control algorithm arises whether, with the new added edges, whether the previously computed set provided by the algorithm remains to control the target (although a more restricted set might indeed become available). In the following we prove that the previous claim is true, namely, our target network control algorithm is indeed robust to false negatives.

Theorem: The TarCoOp target structural controllability algorithm is robust to false negatives.

Proof: Let $G = (V,E)$, $T \subseteq V$, and $D \subseteq V$, be the initial network over the nodes $V = \{1,...,n\}$, the target, and the set of driven (i.e., controlling) nodes, respectively. For the associated matrix representations of the above, let $A \in E^{n\times n}$ be the structural transition matrix corresponding to the network, where each edge is associated an independent variable (by abuse of notation we denote the set of variables also with E) , let $B \in 1_D \times D^n$ be the structural column vector with independent variables $d \in D$ on those positions associated to a driven node in D and 0 otherwise, and let $C_T \subseteq \{0,1\}^{n\times n}$ be the target matrix, i.e., the 0–1 matrix, with $C_T(i,j) = 1$ iff $i = j$ and $i,j \in T$, i.e., C_T is the the identity matrix restricted to the subset T.

As the target T is structurally controllable from D there exists some real values $e_1,...e_{|E|}$ and $d_1,...,d_{|D|}$ for the variables associated to the edges and the variables associated to the input nodes, respectively, such that

$$\text{rank}CoM(A,B,C_T) = \text{rank}[C_T B, C_T AB, ..., C_T A^2 B, ..., C_T A^{n-1} B] = |T|$$

In particular, let $\overline{CoM(A,B,C_T)}^T$ be a $|T| \times |T|$ restriction of $CoM(A,B,C_T)$, such that there exists some non-zero values $k_1,...,k_{|E|,p_1,...,p_{|D|}}$ for the variables $e_1,...e_{|E|}, d_1,...,d_{|D|}$ such that

$$\det(\overline{CoM(A,B,C_T)}^T) = K, \text{ for some } K \neq 0. \tag{3}$$

Assume now that there exists a configuration such that, by adding a few extra edges $\overline{E} = \{\overline{e_1},...,\overline{e_m}\}$, the target T is not any more structurally controllable from D. Let A' be the new structural transition matrix, with inputs over $E' = E \cup \overline{E}$. Thus, we have that:

$$\text{rank}CoM(A',B,C_T) = \text{rank}[C_T B, C_T A' B, , C_T A'^2 B, ..., C_T A'^{n-1} B] < |T|.$$

In particular, it implies that $\det(\overline{CoM(A',B,C_T)}^T) = 0$, for any valuation of the indeterminates $e_1, ...e_{|E|}, \overline{e_1}, ..., \overline{e_m}$ and $d_1, ..., d_{|D|}$ where the $|T| \times |T|$ line and column restrictions over $CoM(A',B,C_T)$ are taken exactly as in $\overline{CoM(A,B,C_T)}$. Let us rewrite the above determinant formula:

$$\begin{aligned} det(\overline{CoM(A',B,C_T)}^T) = f(e_1, ...e_{|E|}, \overline{e_1}, ..., \overline{e_m}, d_1, ..., d_{|D|}) = \\ = g(e_1, ...e_{|E|}, d_1, ..., d_{|D|}) + h(e_1, ...e_{|E|}, \overline{e_1}, ..., \overline{e_m}, d_1, ..., d_{|D|}) = 0, \end{aligned} \quad (4)$$

for some functions $g()$ and $h()$, such that no term in $h()$ depends only on $e_1, ...e_{|E|}, d_1, ..., d_{|D|}$, i.e., such terms are grouped only within g(). Thus, by turning $\overline{e_1} = ... = \overline{e_m} = 0$ we have that $f(e_1, ...e_{|E|}, 0, ..., 0, d_1, ..., d_{|D|}) = g(e_1, ...e_{|E|}, d_1, ..., d_{|D|}) + 0 = det(\overline{CoM(A,B,C_T)}^T)$, and thus, by equation (3) above $g(k_1, ..., k_{|E|, p_1, ..., p_{|D|}} = K \neq 0$.

To conclude, by equation (4) we have that:

$$\begin{aligned} det(\overline{CoM(A',B,C_T)}^T|_{\text{ where } e_1 = k_1, ..., d_{|D|} = p_{|D|}}) = \\ = f(k_1, ...k_{|E|}, \overline{e_1}, ..., \overline{e_m}, p_1, ..., p_{|D|}) = K + h(k_1, ...k_{|E|}, \overline{e_1}, ..., \overline{e_m}, p_1, ..., p_{|D|}) = 0, \end{aligned}$$

for all values of the variables $\overline{e_1}, ..., \overline{e_m}$, which is a contradiction with the fact that each term from $h()$ contains at least one of the variables $\overline{e_1}, ..., \overline{e_m}$.

Robustness to drugs' secondary target effect

The above result on robustness against various uncertainties regarding additional network interactions can be extended to similar uncertainties regarding drug effects over the network's nodes. In the framework of our current research, driven control over a certain node is seen as a certain drug directly affecting the expression level of that protein. However, besides primary/main targets, many drugs are known to have also secondary targets. The question arises whether in case of such drugs having both primary and (potentially even currently unknown) secondary targets, if the output generated by our algorithm (i.e., in the form of a certain set of nodes controlling the target) remains to be a controlling set for the target, in the case when some of the drugs used for manipulating the expression level of these proteins affect also some other nodes within the network.

Indeed, if such un-documented, or simply ignored, interactions of the drugs with other nodes are considered, such interactions are nothing more than additional edges in the initial network. According to our previous result, adding new interactions to the network does not interfere with the properly of a certain set of nodes to control a certain target. (Note however that adding such interactions might indeed interfere with the minimality of the provided control set.)

References

1. Kalman, R. E. Mathematical description of linear dynamical systems. *Journal of the Society for Industrial and Applied Mathematics Series A Control* **1**, 152–192 (1963).
2. CT, L. Structural controllability. *IEEE Trans. Automat. Contr* **19**, 201–208 (1974).
3. Shields, R. & Pearson, J. Structural controllability of multiinput linear systems. *IEEE Transactions on Automatic Control* **21**, 203–212 (1976).
4. Czeizler, E., Gratie, C., Chiu, W. K., Kanhaiya, K. & Petre, I. *Target Controllability of Linear Networks*, 67–81 (Springer Nature, 2016).

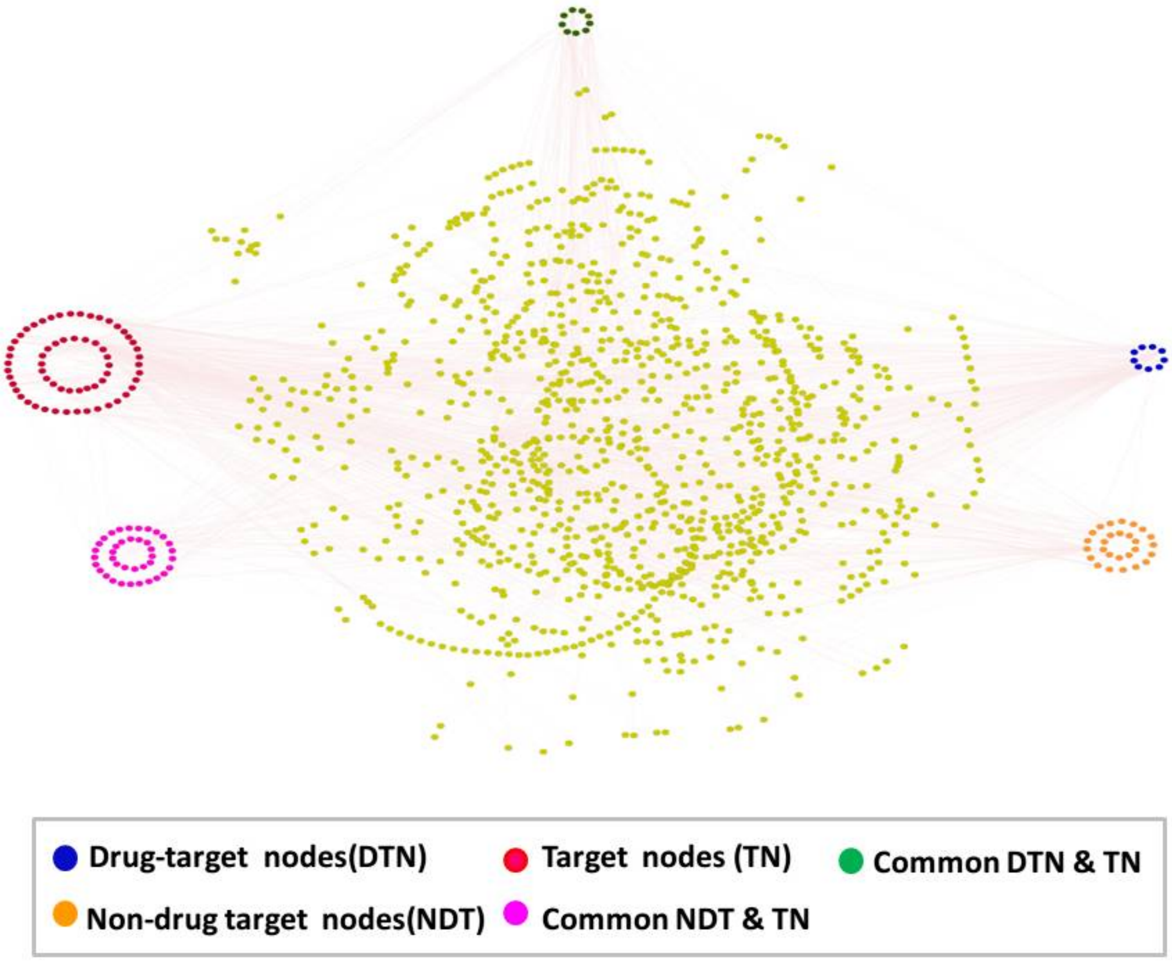

Fig S 1. **Breast cancer PPI network.** A network view of the breast cancer PPI network. This network contains 90% of the total network nodes, while the remaining part of the network containing isolated nodes. The drug-target nodes (DTN) are shown in dark blue, target nodes (TN) are in maroon, nodes that are both in DTN and TN are shown in dark green, non-drug target nodes (NDT) are in light orange, and nodes that both in NDT and TN are in magenta.

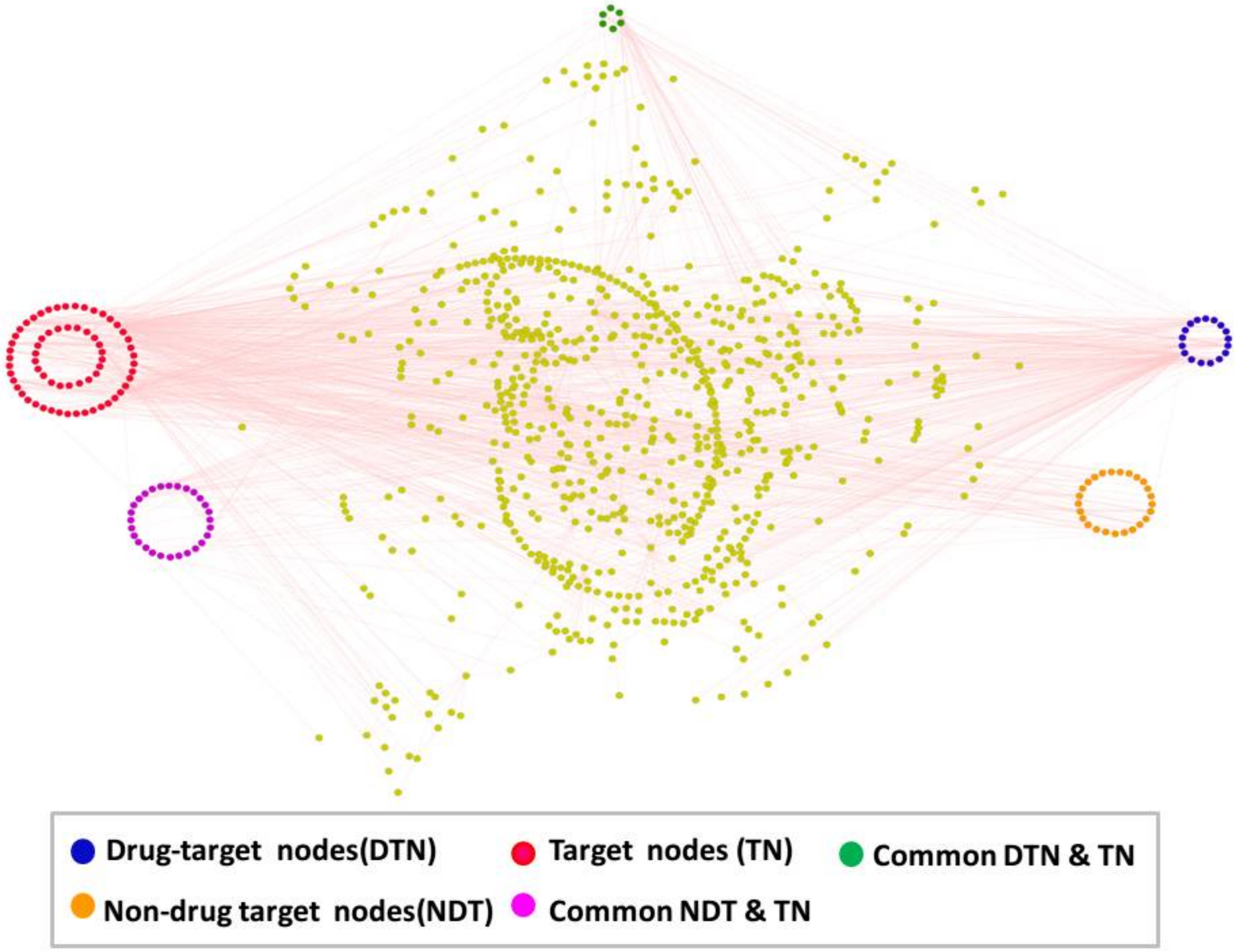

Fig S 2. Ovarian cancer PPI network. A network view of ovarian cancer PPI network. This network contains 90% of the total network nodes, while the remaining part of the network containing isolated nodes. The drug-target nodes (DTN) are shown in dark blue, target nodes (TN) are in maroon, nodes that are both in DTN and TN are shown in dark green, non-drug target nodes (NDT) are in light orange, and nodes that both in NDT and TN are in magenta.
